# Supplementary material for: Chromothripsis during telomere crisis is independent of NHEJ, and consistent with a replicative origin
Source: Genome Res. 2019 May;29(5):737–49. doi: 10.1101/gr.240705.118 (PMC6499312; doi:10.1101/gr.240705.118)
Supplement: Supplemental Material [file supp_gr.240705.118_Supplemental_file_1.zip › contigs/annotated_contigs/DB79/contig.2.DB79_length_820_mean_cov_3.84146341463.docx]

**DB79_length_820_mean_cov_3.84146341463**

```
TCTAGGTGGCTCTCATAGGTGGTTGGGCTAGCGAT|GCCCTCCTCTTGCGGATCAGAAGTGCCGGGTGTGGGTCAGTGTTCTCGGTGGA
                                    >chr9:130321730-130322104 - E=4e-195
GGGTACACGTGCAGGGGTGGGGGCCCAGGGAGTAACTGGGCATCCAGGAGGGGAGGAAGAATGAACTGTGGTTTGCAAAGTGCAGACCC

CACTTTCCCACCTGCCCATCTGTGGCCAAGCCAACTGCAGCTGCTGTCCATCTTTCCCATGCCCCATGCTGAGCAGAACTATTTGTGTC

CAGCCGAATTTGTGAAGCTTAGCACCTTCTAGAAGAGGAGTCCCTTGGGAAGAAGCTGCCCACCCACTCGTGCTCCAGTCACCTCTGAT

TTATTCCACCTGCCAGGAACCACTGGTGGGTGCGGGCTAGGAGGCAGGCTCC|CC|CATCCTAAATTTGCTGGTAGATGACCAAGATTC
                                                     >chr9:97531449-97531862 + E=1e-233
CACCAGTAGAGCTTTATTCAGAGGATAAAACCTTCACACTGCTAAAATGTAGCTACCCAACTGTAGATTGATTTCAGGTTTGCACTGTG

ATGTCAGACATGCATAGGATATCAGCTTGTGAAACAGCAAAAAGCCATTGGTTATAAAGACTCTTAACTCCCTAGCGCTTGGTTTGGGC

ATGTTGGGAAGGGAAGAGTGAGCCTGCAGCTGATGAAGAGAAAAAGGCCAAGAAAAGAATAGGCTGGGAGCACAAAGATAATTTACATT

TTCAAGGTGTGCCTACCCTAAGACCTCCCTCTTATGTGATTGTTTTCCTGCTTTTGAAAGGATTTCAGTAAGAATGTGATTGAGTCTCC

TATCAGACATGGGTATATTAAT
```
